# Delayed hemobilia due to hepatic artery pseudo-aneurysm: a pitfall of laparoscopic cholecystectomy

**DOI:** 10.1186/s12893-016-0175-9

**Published:** 2016-08-22

**Authors:** Mawaddah Alrajraji, Abrar Nawawi, Reda Jamjoom, Yousef Qari, Murad Aljiffry

**Affiliations:** 1 Department of Surgery, Faculty of Medicine, King Abdulaziz University, Jeddah, Saudi Arabia; 2 Department of Medicine, Faculty of Medicine, King Abdulaziz University, Jeddah, Saudi Arabia

## Abstract

**Background:**

Hepatic artery pseudoaneurysm as a complication of laparoscopic cholecystectomy is considered a rare, potentially life threatening condition.

**Case presentation:**

We report a case of late onset hemobilia presenting 8 months following elective laparoscopic cholecystectomy with complex biliary and vascular injury. The patient was treated surgically with primary repair of the aneurysm and hepaticojujenostomy.

**Conclusion:**

A high index of suspicion should be raised when encountering a patient with massive upper GI bleeding and a previous history of hepatobiliary manipulation or surgery regardless of postoperative period.

## Background

Hemobilia and vascular injuries are among the most important - albeit not necessarily the most common- complications of laparoscopic cholecystectomies (LC), due to the high morbidity and mortality associated with the condition [1–3]. The symptoms of hemobilia commonly appear within the early postoperative period or as late as 4 weeks [3]. Alongside with a detailed literature review, we report a case presenting 8 months after LC with right hepatic artery pseudo aneurysm in association with a complex injury to the common bile duct. To our knowledge there are two similar cases in the literature reporting a delayed hepatic artery pseudoanyrusm presenting up to a year following LC [4, 5] (Table 1).

**Table 1:** Summary of similar cases (post laparoscopic cholecystectomy hepatic artery pseudoaneurysm) reported in the literature

Author	Age	Gender	Presentation	diagnosis	Time of presentation	Procedure	Outcome
Genyk YS [18]	57 years	F	Pain, UGIB and jaundice.	HPA	2 weeks	Embolization	2 year follow up
Jean-Denis Yelle, et al. [19]	48 years	F	Pain, UGIB.	Contrast study of the fistula, ERCP& HPA	NA	Open laparotomy	6 month
Siablis D, et al. [7]	29 years	M	Pain, jaundice and UGIB.	HPA	NA	Embolization	Close follow ups
Sam T.M. Kwauk, et al. [20]	39 years	F	N\V, pain and jaundice.	CT and HPA	NA	Selective embolization	4 month
Ribeiro A, et al. [4]	57 years	F	Pain	UGI endoscopy, CT and HPA	13 months	Emergent laparotomy.	NA
de Blaauw I, et al. [21]	38 years	F	Pains and melena	NA	NA	Emergent laparotomy.	7 month
T nicholoson et al. [22]	43 years	F	Hematemesis	NA	43 days	Embolization	Well at 15 month
	69 years	M			10 days		Well at 5 years
	54 years	F			5 days		Well at 7 years
	42 years	F			8 days		Well at 4 years
	65 years	F			18 days		Well at 6 years
	47 years	F			6 days		Well at 3 years
	39 years	M			7 days		Well at 5 years
	68 years	F			9 days		Well at 2 years
	53 years	F			12 days		Well at 6 years
Dogru O, et al. [23]	62 years	F	UGIB	Ultrasound and UGI endoscopy	NA	Exploratory laparotomy	NA
Iannelli A et al., [24],	36 years	F	Not mentioned	NA	NA	Selective embolization	NA
G Roche-Nagle, et al. [25]	58 years	F	Pain, and vitally collapsed	CT & HPA	NA	Exploratory laparotomy	Uneventful recovery.
Mandur Ma et al. [15],	57 years	M	UGIB	NA	2 weeks	Embolization	Well at 22 month
	63 years	F	UGIB	NA	4 weeks	Embolization	Well at 12 month
	54 years	M	UGIB	NA	3 weeks	Ligation	Well at 6 months
Nakase Y, et al. [26]	63 years	F	Pain and UGIB	HPA	NA	Selective embolization	NA
Masannat YA [27]	71 years		Not mentioned	Angiogram	NA	Coil embolization	NA
Srinivasaiah N [28]	57 years	M	Pain and hematemesis	Ultrasound, CT and HPA	4 weeks	Radiological intervention	NA
Yao CA, et al. [29]	54 years	M	Pain, UGIB, and disturbed LFTs.	CT.	NA	Angiography with embolization	NA
Sansonna et al., [30]	44 years	F	UGIB	CT	3 weeks	Angiography with embolization	Well at 2 weeks
Paseka T et al., [31]	51 years	M	UGIB	CT & HPA.	Months	Exploratory laparotomy	Well at 6 months.
AD Mate et al., [32]	45 years	M	LGIB	HPA	15 days	Emergency laparotomy	NA
El Bouhaddouti, et al. [33]	50 years	M	Pain, jaundice and UGIB	UGI endoscopy & HPA	3 months	Emergent laparotomy.	1 year
Thamer A. Bin Traiki et al. [34]	65 years	M	Febrile (38.9 °C, (left brachial vein Thrombosis).	CT, ERCP, & HPA.	4 weeks	Angiography and embolization.	well at discharge
Tun-Abraham ME et al., [35]	67 years	M	Biliary leakage, sepsis and late intra-abdominal bleeding	CT.	NA	Angiography with embolization	No evidence of recurrent bleeding
Abdallah S et al., [36]	40 years	M	Obstructive jaundice and pain	CT & HPA.	NA	Selective embolization	NA
Chih yang Hsiao et al., [37]	40 years	M	Jaundice, pain and oozing blood from drainage.	MR & HPA.	2 weeks	Embolization and angiography.	NA

*Abbreviation definition*: *NA* not available, *UGIB* upper gastrpintestinal bleeding, *HPA* hepatic selective angiography, *ERCP* endoscopic retrograde cholangiography, *LC* laparoscopic cholecystectomy

## Case presentation

A 41-year-old female patient presented to our emergency department with history of upper gastrointestinal (UGI) bleeding in the form of painless coffee ground vomitus and melena. Patient has no significant past medical history apart from uneventful elective laparoscopic cholecystectomy due to a remote episode of acute cholecystitis in another institution 8 months prior to her presentation. Upon reviewing the patient’s charts, the operation was smooth, no intraoperative complications encountered, monopolar cautery energy source was used and no intraoperative cholangiogram was obtained.

The patient had history of previous attack of minimal UGI bleeding 6 weeks post cholecystectomy, at that time an upper endoscopy and ERCP were done showing hemobilia, and a stent was placed in the common bile duct. Following that, the patient was relieved of symptoms and a CT study confirmed the presence of a small (<0.25 cm) right hepatic artery pseudoaneurysm. The patient was offered the option of embolization, however she refused the treatment and lost follow up until the current presentation.

Upon her presentation to our center, the patient was pale, tachycardiac (100–110 bpm) and normotensive. Abdomen was soft and lax with no sign of peritonitis were noted upon palpation.

## Laboratory results

Hemoglobin: 10.3 g\L, Hematocrit: 33, Platelets: 44 × 10^9^/L. *Coagulation profile was normal and Liver function showed a mild elevation of the liver enzymes*.

**Fig. 1:**
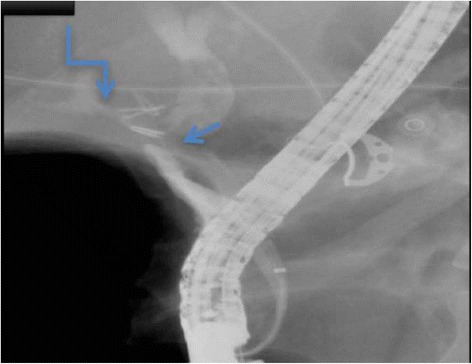
ECRP study: showing the stricture of the CBD (*straight arrow*), with filling defect of the CHD and contrast filling the right hepatic artery communication between hepatic artery and CHD (*angulated arrow*)

**Fig. 2:**
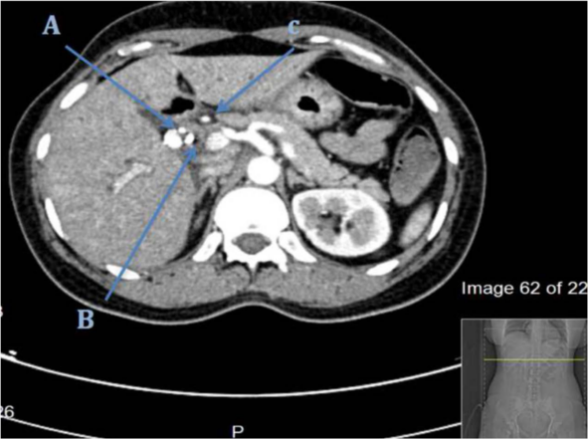
CT study, an axial cut: the arrows points at, A: right hepatic artery psuedoaneurysm B: Stent C: common hepatic artery

**Fig. 3:**
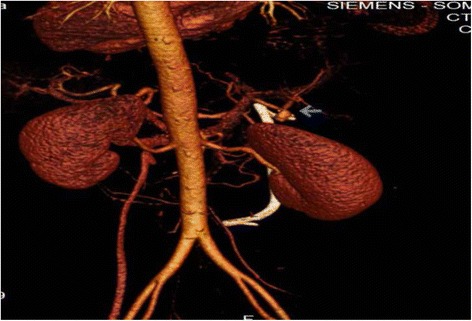
3D angiogram demonstrating aneurysm of hepatic artery (*straight arrow*)

The patient was hospitalized and resuscitated, after which she was prepared for an emergency *UGI* endoscopy, where no bleeding source was identified in the stomach or duodenum. However, blood was noticed flowing from the major duodenal papilla raising suspicion of hemobilia. On ERCP the old stent was dislodged and a stricture was identified at the common bile duct (CBD) extending into the hepatic duct but below the bifurcation. In addition, the bile duct was filled with clots and the right hepatic artery started to fill with contrast. A plastic stent was placed across the stricture (Fig. 1). CT abdominal angiography was done showed saccular lesion at the right hepatic artery suggestive of the presence of pseudo-aneurysm (1.5 cm) (Figs. 2 and 3). Bleeding was initially controlled following the ERCP (stenting). Shortly after, the patient deteriorated again, with a drop of her hemoglobin to 7.7 g\L, she was transferred to the critical care unit, resuscitated with 4 units of PRBCS and platelets. Patient was hemodynamically stable and referred for angiography for angio-embolization, which was not successful due to failure to cannulate the common hepatic artery. The patient continued to experience gastrointestinal bleeding requiring further transfusion of blood products. She was taken to the operating room for an emergency exploratory laparotomy.

### Operative note

**Fig. 4:**
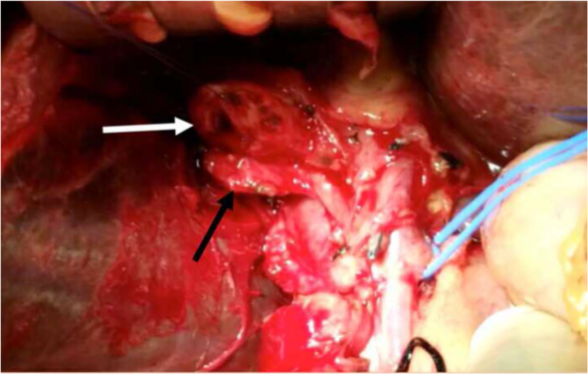
Intra-operative: the arrows points at, A: (*white*) Common bile duct, B: (*black*) Right hepatic artery

Exploration of the abdomen was obtained through a midline laparotomy; there was no evidence of heamoperitonium. After obtaining proximal control of the common hepatic artery, isolation of right hepatic artery was difficult. The area of the porta hepatis was thickened and edematous probably due to previous cholangitis and ERCPs. The CBD was divided to facilitate the exposure of the right hepatic artery. Following that, the right hepatic artery course was isolated and controlled proximal to the pseudo-aneurysm (Fig. 4). Upon dissection the aneurysm was accidently opened with minimal back bleeding. The wall of the pseudo aneurysm was refreshed and the right hepatic artery was closed in a primary repair fashion.

Roux-en-y hepatico-jujenostomy was performed. The stent has migrated and it was felt in the transverse colon, it was retrieved through a colotomy.

The patient had an uneventful recovery apart from wound infection. Her LFT gradually returned back to normal levels. After discharge the patient had been followed up for 14 months with no evidence of reoccurrence of bleeding.

## Discussion

Laparoscopic cholecystectomy (LC) carries the risk of biliary and vascular iatrogenic injuries even in the hands of most experienced surgeons. Despite its many benefits in comparison to open procedure it carries a 10-fold increased risk of iatrogenic biliary and vascular injuries [3]. Laparoscopic biliary tree injuries are reported in 0.3–1 % of procedures, whereas vascular injuries reported in 0.25–0.5 % (7–9). It is well known in the literature that intraoperative bleeding is the most common manifestation of LC iatrogenic arterial injury followed by ligation. A less common manifestation of post LC iatrogenic arterial injury is presented here.

Approximately 10 % of all the reported cases of hemobilia are secondary to iatrogenic hepatic artery pseudo-aneurysms (Table 1) (8).

Upper GI hemorrhage is the most common presentation of a ruptured right hepatic artery pseudo-aneurysm into the biliary tract. However, the classic presentation of hemobilia known as Quinke’s triad is seen in less than 40 % of patients [1, 6, 7].

To date, the definite pathological explanation of post LC hemobilia is still unclear but suggested mechanisms are mechanical, thermal injuries specially monopolar coagulation during laparoscopic surgeries and surgical clips encroachments [8–10]. Bile leak and superimposed infection are important precipitating factors, It has been reported that bile acid could contribute to the injury to the vascular wall resulting in delayed healing of the vessel wall which leads to the development of pseudoaneurysm [11]. We believe the mechanism of injury in our patient is thermal, due to the presence of vascular and biliary injuries and her delayed presentation.

There are several options for diagnosing and managing such condition, Upper gastrointestinal endoscopic evaluation is fundamental to exclude the more common causes of UGI bleeding [12]. In the current Literature nearly 12 % of cases reported diagnosed endoscopically [13].

Contrast enhanced computed tomography of the abdomen aids in determining the diverse etiological causes of the hemobilia [14]. The difficulty in making the diagnosis of hemobilia might be attributable to the fact that the bleeding is usually intermittent.

The management of hemobilia is an acute emergency as patient might exsanguinate when ruptured. The therapeutic aim is to stop the bleeding and to relive biliary obstruction [13]. Transarterial embolization (TAE) is the treatment choice for all causes of hepatic artery aneurysm with a high rate of success, surgical intervention should be done for selected patients who fail a trial of embolization (12) as in our case. Angiography offers the advantage of minimally invasive procedure, and it also represents an effective treatment choice for this potentially fatal complication [15, 16].

Surgery is narrowed to conditions requiring; bile duct repair, extra-hepatic lesion or gallbladder hemorrhage, and for failure of TAE [15, 17].

The time phase between confirming the diagnosis of hemobilia and the decision for surgical intervention in case of absence or failure of embolization is crucial and must be managed meticulously by the attending surgeon. As these patients are at risk of sudden rupture and exsanguination.

## Conclusion

A high index of suspicion should be considered by all treating surgeons when encountering instances of hemobilia in patients presenting with upper GI hemorrhage with a past history of cholecystectomy regardless of the post-operative period. Assessment of the hepatic arteries is an important aspect of the investigation of all biliary injuries. Intraoperative preventive measures are paramount in preventing these complications, such as careful dissection and the avoidance of cautery usage adjacent to the vasculo-biliary structures during LC.
